# Integrating Machine Learning into Additive Manufacturing of Metallic Biomaterials: A Comprehensive Review

**DOI:** 10.3390/jfb16030077

**Published:** 2025-02-21

**Authors:** Shangyan Zhao, Yixuan Shi, Chengcong Huang, Xuan Li, Yuchen Lu, Yuzhi Wu, Yageng Li, Luning Wang

**Affiliations:** 1 Beijing Advanced Innovation Center for Materials Genome Engineering, School of Materials Science and Engineering, University of Science and Technology Beijing, Beijing 100083, China; d202410300@xs.ustb.edu.cn (S.Z.); d202210281@xs.ustb.edu.cn (Y.S.); d202310310@xs.ustb.edu.cn (C.H.); d202410299@xs.ustb.edu.cn (X.L.); luyuchen0420@foxmail.com (Y.L.); m202310373@xs.ustb.edu.cn (Y.W.); luning.wang@ustb.edu.cn (L.W.); 2 Institute of Materials Intelligent Technology, Liaoning Academy of Materials, Shenyang 110004, China

**Keywords:** additive manufacturing, machine learning, biomaterials, pre-processing design, processing optimization

## Abstract

The global increase in osteomuscular diseases, particularly bone defects and fractures, has driven the growing demand for metallic implants. Additive manufacturing (AM) has emerged as a transformative technology for producing high-precision metallic biomaterials with customized properties, offering significant advantages over traditional manufacturing methods. The integration of machine learning (ML) with AM has shown great promise in optimizing the fabrication process, enhancing material performance, and predicting long-term behavior, particularly in the development of orthopedic implants and vascular stents. This review explores the application of ML in AM of metallic biomaterials, focusing on four key areas: (1) component design, where ML guides the optimization of multi-component alloys for improved mechanical and biological properties; (2) structural design, enabling the creation of intricate porous architectures tailored to specific functional requirements; (3) process control, facilitating real-time monitoring and adjustment of manufacturing parameters; and (4) parameter optimization, which reduces costs and enhances production efficiency. This review offers a comprehensive overview of four key aspects, presenting relevant research and providing an in-depth analysis of the current state of ML-guided AM techniques for metallic biomaterials. It enables readers to gain a thorough understanding of the latest advancements in this field. Additionally, the this review addresses the challenges in predicting *in vivo* performance, particularly degradation behavior, and how ML models can assist in bridging the gap between *in vitro* tests and clinical outcomes. The integration of ML in AM holds great potential to accelerate the design and production of advanced metallic biomaterials.

## 1. Introduction

Metal additive manufacturing (AM) is an advanced technology that utilizes metal powder melting and rapid solidification molding to create intricate structures with high precision [1]. Compared to traditional subtractive (e.g., turning, grinding) and formative manufacturing methods (e.g., casting, extrusion, welding), AM offers superior machining precision, reduced time costs, and a broader selection of materials, contributing to its growing prominence in various industries. In the medical field, metal AM has shown significant promise [2,3], particularly in the production of orthopedic implants [4] and vascular stents [5]. While non-degradable titanium-based alloys [6] and cobalt chromium alloys [7] have traditionally been the materials of choice due to their excellent mechanical properties and established clinical use, these materials pose challenges, such as hindering bone tissue regeneration and causing inflammation. In recent years, biodegradable metals like magnesium (Mg)-based alloys, zinc (Zn)-based alloys, and iron (Fe)-based alloys have garnered increasing attention as promising next-generation materials for implants, offering the potential for improved patient outcomes and reduced long-term complications [8,9,10,11,12].

However, the AM process involves complex multi-physical interactions and high-dimensional calculations, making real-time control and process optimization challenging. The traditional approach of parameter testing, while effective, can be time-consuming and costly. To address these challenges, data-driven methods have been introduced into AM, facilitating process and performance optimization [13]. Machine learning (ML), as a branch of artificial intelligence (AI), leverages real-time data from the AM process and applies algorithmic models to optimize manufacturing, improve performance, and reduce costs [14]. Notably, ML has shown great potential in replacing experimental trials with simulations based on high-confidence predictive models, offering substantial time and cost savings [15]. 

As the demand for customized metallic biomaterials grows, driven by the need to meet the specific requirements of individual patients, the role of ML becomes even more critical. The ability to fine-tune alloy compositions and structural designs can significantly enhance the mechanical properties and degradation behavior of implants [16,17,18]. ML can aid in selecting optimal components and designing structures that meet precise performance criteria, further advancing the field of biomaterials.

The integration of ML in the AM of metallic biomaterials holds significant potential and academic value. This review provides a comprehensive exploration of ML-guided AM for metallic biomaterials, beginning with foundational concepts and advancing to more sophisticated applications. It also highlights several key studies that offer valuable insights. Additionally, this review outlines promising future directions for applying ML to the biological research of AM-processed metallic materials.

For this review, an extensive literature search was conducted using keywords such as ‘additive manufacturing’, ‘machine learning’, and ‘biomaterials’ across databases including Springer, Google Scholar, Elsevier, and PubMed. The majority of the papers reviewed were published between 2017 and 2025, with several key references dating earlier. In total, more than 110 articles were reviewed.

## 2. AM Techniques

**Figure 1:**
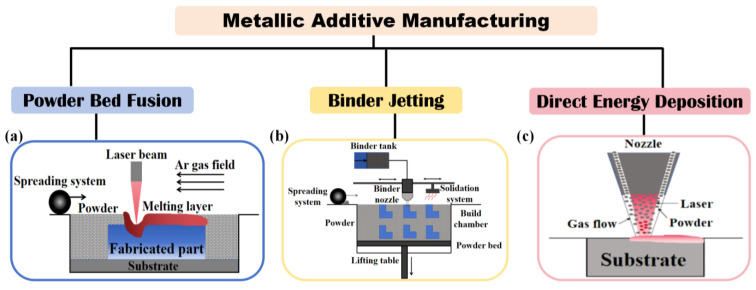
Schematic diagram of mainstream metallic AM methods: (**a**) powder bed fusion; (**b**) binder jetting; (**c**) direct energy deposition, respectively.

AM, which fabricates components using sliced computer-aided design (CAD) models, enables near-net shape production through the rapid melting, cooling, and solidification of metal powder layers. Metal AM stands out for its high material utilization, design flexibility, precision, and environmental advantages, making it a key technology in industries such as aerospace, biomedicine, and equipment manufacturing [19,20,21]. The primary metal AM techniques include powder bed fusion (PBF), binder jetting (BJT), and direct energy deposition (DED) (Figure 1).

### 2.1. Powder Bed Fusion (PBF)

PBF employs a high-energy laser or electron beam in combination with a powder bed system to selectively melt and deposit material layer by layer along a predetermined path. This process generates high-temperature gradients and rapid cooling rates, which refine the grain structure but also introduce significant residual stresses. PBF techniques include selective laser melting (SLM) and electron beam melting (EBM). While rapid cooling enhances mechanical properties through fine grain strengthening, the substantial residual stresses from energy input can initiate cracks and reduce ductility. Consequently, achieving an optimal microstructure and high relative density in the final specimen remains a critical goal in PBF manufacturing. Extensive research has investigated the influence of processing parameters on microstructure formation and their subsequent impact on the mechanical properties of PBF-manufactured components. Studies have also focused on optimizing parameters for specific powders [22,23,24,25,26] and employing heat treatment strategies to further improve the performance of PBF specimens [27].

### 2.2. Binder Jetting (BJT)

BJT involves spraying a binder onto a powder bed to selectively bind powder particles, forming the desired shape. This process operates at room temperature, enabling the production of complex structures without requiring high energy input. BJT is compatible with metal, polymer, and ceramic materials and is particularly advantageous for manufacturing parts with high laser reflectivity. Unlike other additive manufacturing techniques, BJT does not require support structures, and the absence of melting or sintering eliminates residual stress, contributing to enhanced mechanical properties of the fabricated parts [28]. BJT integrates several technologies, with a focus on powder selection, layer formation, and post-processing. Producing high-quality BJT parts depends on critical factors such as powder fluidity, binder viscosity and burnout characteristics, the optimal binder saturation level, and the sintering process [29,30,31,32]. This technique has been widely adopted for creating casting molds with high geometric freedom. Current research aims to expand production scales, both in terms of part size and the number of defect-free specimens with tailored material properties [33].

### 2.3. Direct Energy Deposition (DED)

DED integrates a laser source, gas channel, and powder feed system into a unified process, making it highly efficient for repairing and augmenting defective parts. DED provides flexibility in tailoring thermal properties by adjusting the raw material composition, which is particularly advantageous for fabricating components designed for harsh environments [34]. With high material deposition rates and the ability to process relatively coarse powders, DED enables rapid production and cost reduction [35]. A key strength of DED is its suitability for producing functionally graded materials, facilitated by its *in situ* powder-mixing capability. However, the resolution of DED is generally lower than that of selective laser melting (SLM), necessitating precise control of time-varying parameters and post-processing to achieve desired properties [36]. Current research focuses on improving *in situ* powder mixing and monitoring systems [37,38,39], investigating the thermal dynamics of molten pool evolution [40,41], and eliminating the formation of brittle intermetallic phases [42].

## 3. AM for Metallic Biomaterials

**Figure 2:**
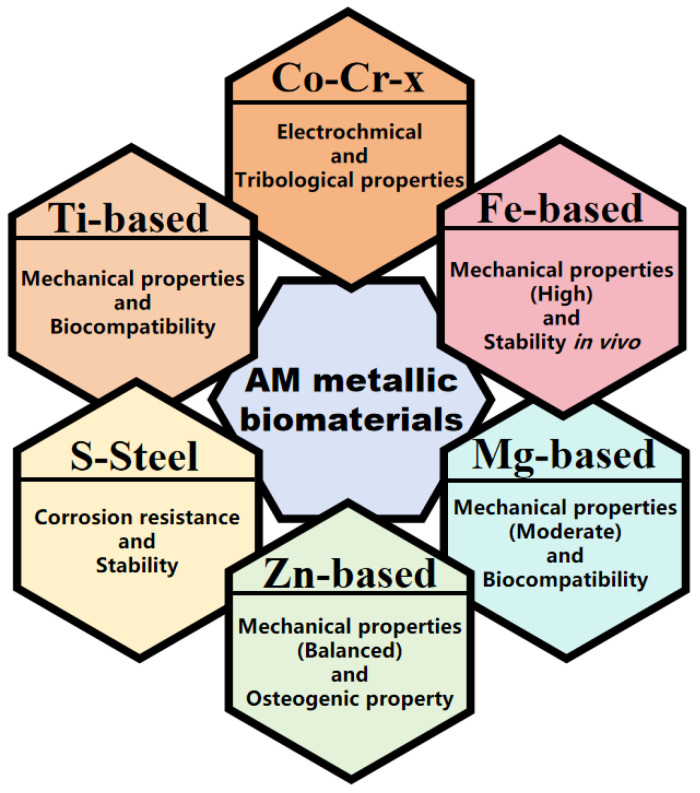
Typical AM biomaterials and characteristics.

AM is highly adaptable for the production of implants with specific shapes or porosities, making it suitable for both bioinert implants and biodegradable scaffolds (Figure 2). 

### 3.1. Bioinert Metallic Materials

Implants such as knee and hip replacement prostheses, which require long-term *in vivo* use, must exhibit excellent corrosion resistance. As a result, inert metallic materials have garnered significant attention. Titanium (Ti)-based alloys, cobalt–chromium (Co–Cr) alloys, and stainless steel (SS) are commonly utilized in the fabrication of orthopedic implants and vascular stents [43,44]. 

Ti-based bone substitutes, known for their exceptional mechanical properties and biocompatibility, are widely applied in biomedical fields. For example, porous Ti-based jaw scaffolds fabricated via electron beam melting (EBM) were tested in transplantation experiments involving dogs with mandibular defects. The results showed that the grafted mandibles closely resembled native mandibles 12 months post-operation, highlighting their potential for human jaw repair [45]. Similarly, clinical trials of EBM-fabricated porous Ti-based acetabular implants, with an average follow-up of 48.2 ± 3.6 months, demonstrated superior survival rates, clinical efficacy, and biological fixation [46]. 

Co–Cr alloys, typically containing 25–30 wt.% chromium and alloyed with molybdenum and tungsten to enhance wear resistance, are considered suitable candidates for biological implants [47]. The layer-by-layer thermal history of AM results in a microstructure resembling weld pool boundaries, characterized by a predominantly columnar grain structure aligned along the build direction [48]. Studies have shown that the primary phase in AM Co–Cr alloys is the metastable face-centered cubic (FCC-γ) phase, which can persist below the solvus temperature [49] and significantly influence mechanical properties [50,51]. The electrochemical, tribological and biological properties of these alloys have been extensively investigated [52,53,54,55]. Multicomponent AM Co–xCr–yMo–zW alloys are believed to offer distinct advantages over cast Co–Cr alloys as biomaterial implants.

SS generally exhibits poorer biocompatibility and osteointegration compared to titanium alloys. Some studies have reported allergic reactions associated with SS stents [56]. Nonetheless, SS remains advantageous in terms of reducing corrosion risks and manufacturing costs, which are still utilized in certain applications in oxygen-rich environments including arteries and the oral cavity [57]. AM stainless steel dental implants have demonstrated excellent mechanical and geometric properties without requiring extensive manual pretreatment or post-treatment. Furthermore, the analysis of assembly clearances has demonstrated a considerable level of medically acceptable accuracy [58]. 

### 3.2. Biodegradable Metallic Biomaterials

In recent years, biodegradable materials for applications such as vascular stents, orthopedic implants, and contraceptive devices have garnered significant attention [59]. These materials are designed to degrade within the body over time, ideally aligning with the healing process. To achieve the optimal mechanical properties and degradation rates, various multicomponent alloys have been developed, with magnesium (Mg)-based, iron (Fe)-based, and zinc (Zn)-based alloys being the most extensively studied.

Mg-based alloys are particularly appealing due to their mechanical properties, which closely resemble those of natural bone, effectively minimizing the stress shielding effect during bone recovery [60]. However, their high degradation rate poses challenges in meeting the required service life in the human body, and trace impurity elements can elevate the risk of localized corrosion [61]. Additionally, the wide solidification temperature range, low eutectic temperature, and high thermal stresses associated with AM processes increase the likelihood of solidification cracking in deposited layers, with repeated heating further exacerbating this issue [62]. Research has shown that factors such as sample size, preheating conditions, and scanning strategies must be carefully optimized to mitigate defects in AM Mg-based alloys, thereby improving their viability for biomedical applications [63,64].

Fe-based offer superior mechanical properties compared to Mg-based alloys and exhibit greater stability within the human body over extended periods [65]. However, the slow degradation rate of Fe-based alloys can hinder bone regeneration, and residual materials may necessitate surgical removal, posing risks of secondary harm to patients. The corrosion behavior of Fe-based alloys is influenced by factors such as grain boundary volume, pit-induced impurities from severe deformation, and the homogenization of non-metallic phases. These factors impact the formation and stability of the passive film, making the corrosion rate challenging to control [66]. 

Zn-based alloys, on the other hand, possess a moderate degradation rate, which falls closer to the ideal range for biodegradable metals, offering a balanced combination of corrosion resistance and mechanical properties [67]. Recent studies underscore the advantages of Zn-based alloys, highlighting their ability to alleviate the stress shielding effect [68] and promote bone cell growth and differentiation [69]. The properties of AM Zn-based alloys can be tailored through the addition of various elements [70,71,72]. Despite their potential, the development of AM Zn-based alloys faces challenges during sample preparation due to their low melting temperature. This characteristic leads to significant evaporation during processing, resulting in issues such as splashing and porosity, which compromise print quality [73]. 

### 3.3. Challenges of AM Metallic Biomaterials

Despite significant progress in AM for metallic biomaterials, several challenges continue to hinder its practical application. The selection of raw materials for AM remains highly diverse, with alloy composition regulation largely reliant on prior experience. Developing multicomponent alloys often necessitates complex phase diagram calculations. Additionally, optimizing AM processes is challenging due to the numerous interdependent parameters, such as input energy density and environmental conditions, which frequently require costly trial-and-error approaches. Simplifying parameter optimization and enabling *in situ* monitoring of forming quality are therefore critical areas of focus. Structural design in AM also presents notable challenges. The lack of advanced methodologies for creating intricate porous structures has constrained the application of such implants. Consequently, there is an urgent need for efficient and robust structural design techniques. Addressing these challenges at both the design and processing levels necessitates the development of intelligent manufacturing systems capable of optimizing the entire AM workflow. These systems hold the potential to revolutionize AM by enhancing efficiency, precision, and scalability.

## 4. Machine Learning in AM

*In situ* physical signals and characterization data, encompassing optical [74], acoustic [75], thermal [76], microstructure morphology [77], and mechanical properties [78], have shown considerable potential in optimizing AM techniques. However, the AM process presents analytical challenges due to the coupling of multiple physical fields and the complexity of high-dimensional computations, underscoring the need for efficient data integration methods. The advancement of artificial intelligence (AI) has positioned machine learning (ML) as a powerful tool for the integration and analysis of complex datasets. As a result, a diverse range of ML algorithms is being progressively incorporated into AM research and applications.

### 4.1. Brief Introduction of ML

ML, a subfield of AI, is focused on enhancing the autonomous learning capabilities of computer systems. By leveraging existing data patterns and experiences, ML eliminates the need for explicit programming. At its core, ML involves data-driven model training, which enables these models to predict or make decisions based on new, unseen datasets. Through the integration of intelligence into software systems, ML has significantly advanced the development of automated AI capabilities [79]. 

The roots of ML trace back to the 1940s, with pioneering research on artificial neural networks [80]. Early mathematical models were employed for tasks such as data clustering and dimensionality reduction, streamlining the analysis of complex datasets. However, these initial algorithmic developments were fragmented and lacked a unified framework. By the 1980s, a surge in innovation and integration of algorithms catalyzed the widespread growth of ML research. Marvin et al. [81] articulated a foundational principle of ML, emphasizing the importance of improving algorithm usability and reducing computational complexity.

ML encompasses a variety of classifications based on training methodologies and the types of data involved. These classifications can be tailored and optimized to meet the specific requirements of diverse application scenarios.

### 4.2. Categorization of ML Methods

**Figure 3:**
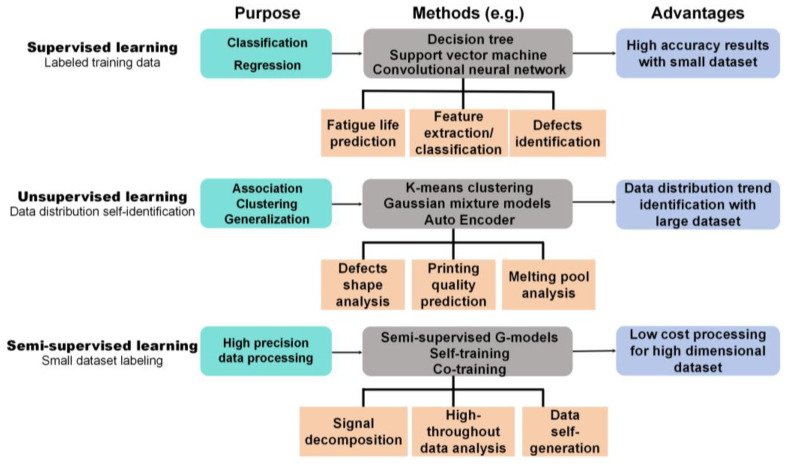
Basic introduction of three machine learning categories.

ML methods are generally classified into three categories: supervised, unsupervised, and semi-supervised learning (Figure 3).

Supervised learning involves training a model on labeled data, where the accuracy of the algorithm is evaluated through error calculations. By identifying the characteristics of the training data, supervised learning is particularly effective for tasks such as data classification and feature extraction. The process parameters of AM exhibit a strong correlation with the final quality of fabricated specimens, as the manufacturing process significantly influences the microstructure and density, thereby affecting mechanical properties. Supervised learning models, when provided with precise labeled data, demonstrate high predictive and classification accuracy, which enable the direct determination of printing quality and further assist in judging the optimal process parameters. Supervised learning offers a compelling approach for identifying process-performance factors in AM. Furthermore, AM-associated online (*in situ* physical signals) and offline (subsequent characterization results) information can be leveraged to establish these models. In AM, supervised learning has proven beneficial for process optimization, with several studies using supervised models for feature classification and defect identification. Hermann et al. [82] previously proposed that the defects of AM specimens were detected with destructive component tests after process, which had already resulted in time loss and increased the workload of operators. An ML architecture based on convolutional neural network and *in situ* thermographic imaging was established, which can be used to identify delamination defects and splatters. The model correctly identified the vast majority of delaminations and splashes. Furthermore, this model can evaluate the entire image instead of a certain pixel, which made it possible to detect large defects. In another study, Zhang et al. [83] proposed that optical emission signals generated during the AM process encapsulated substantial information pertaining to the molten pool, which is critical for characterizing molten pool dynamics. However, these optical signals are frequently intertwined with extraneous signals and contaminated by noise, often resulting in the loss of critical information during denoising and filtering procedures. Notably, research on the integration of multi-source signal data remains limited. To address this gap, they implemented a machine vision-based monitoring system capable of simultaneously capturing molten pool, plume, and spatter information. They extracted distinctive signal features from single-track AM specimens with varying quality levels and employed multiple ML algorithms to classify these features. The results revealed that the convolutional neural network effectively learned discriminative features from the fused multi-modal signals and achieved superior classification accuracy, which was well-suited for *in situ* monitoring and judging optimal laser parameters. Several commonly used supervised learning methods along with their principle and respective advantages are listed in Table 1.

Unsupervised learning, in contrast, operates without predefined labels. Instead, it classifies data by minimizing intra-class variation and maximizing inter-class variation. Unsupervised learning finds significant application in additive manufacturing (AM), particularly in defect identification within laser-based AM processes. The energy input during AM generates diverse molten pool geometries and porosity, directly influencing the surface roughness and density of fabricated components. Unsupervised learning is well-suited for large datasets, enabling the extraction of patterns, structures, and features from unlabeled data while minimizing labeling costs. Compared to microstructural morphology, molten pool and pore characteristics are more accessible, facilitating the cost-effective construction of large datasets and serving as ideal inputs for unsupervised learning. Developing unsupervised learning models to optimize process parameters is a promising strategy for advancing the adoption of AM technologies. Hertlein et al. [84] developed a Bayesian network (BN) in a continuous domain based on the relationships between certain AM process parameters and specimen quality characteristics. The authors extracted data from a large number of publications on SLM-SS316L to train the BN. The network incorporates four process parameters (laser power, scan speed, hatch spacing, and layer thickness) and five quality characteristics (density, hardness, top surface roughness, ultimate tensile strength in the build direction, and ultimate tensile strength perpendicular to the build direction). By de-emphasizing critical parameters such as laser type, spot size, preheating, and powder diameter, the BN ensures the model’s applicability across different SLM machines, making it a robust parameter-quality prediction model with strong generalization capabilities. Snell et al. [85] emphasized that variations in laser energy input conditions can induce distinct types of porosity defects in the fabricated specimens. Insufficient energy input leads to incomplete melting of the metal powder, thereby generating lack-of-fusion (LF) defects, whereas excessive energy input causes over-penetration of the molten material, resulting in the formation of keyhole-induced (KH) defects. Therefore, the detection and characterization of defects in the final product can serve as a critical diagnostic criterion for AM operators to evaluate and optimize laser parameters. A K-means clustering method was trialed on 3D X-CT pore geometric data, which was successfully applied to the classification of LF and KH pores. This work provides valuable guidance for a deeper understanding of pore formation under different laser conditions. Several unsupervised learning methods are listed in Table 2.

Semi-supervised learning combines elements of both supervised and unsupervised learning. This method uses a small subset of labeled data alongside a larger unlabeled dataset to train models. By leveraging multiple ML techniques, semi-supervised learning enables efficient data processing and model building, balancing accuracy with cost-effectiveness. The quality of AM specimens are contingent upon multiple factors. Limited datasets may fail to comprehensively represent the entire parameter space, while large datasets entail significant costs. Semi-supervised learning, integrating the advantages of both supervised and unsupervised learning, mitigates the expense of labeled data acquisition, which is versatile across tasks such as classification, regression, and clustering. It is considered a promising direction for advancing machine learning-assisted AM. This approach is particularly valuable in scenarios where labeled data are limited but high learning accuracy is required. Okaro et al. [86] argued that during the optimization of AM parameters, process measurements (labels) correlated to final quality are generated whenever a specimen is manufactured. However, cost constraints often prevent the assignment of ’labels’ to the majority of this data. To address this challenge, a semi-supervised Gaussian mixture model (GMM) was developed. The model was applied to a large amount of AM process data with few labeled data. The optical signals generated in tensile bars fabrications via PBF were captured by a photodiode system, which were subsequently subjected to feature extraction and utilized as training data to develop the model. A comprehensive dataset comprising 49 data points was utilized and applied by a 2-fold cross-validation strategy. The semi-supervised learning method was systematically compared with the supervised method. Validation results revealed that the semi-supervised method achieved a superior average percentage success rate. Particularly, as the ratio of unlabeled training data to labeled training data increased, the semi-supervised method demonstrated progressively more pronounced advantages. Several semi-supervised learning methods are listed in Table 3.

### 4.3. ML in Alloy Composition Design

Multicomponent alloys exhibit complex solidification kinetics, with real-time compositional changes during the AM process, leading to variations in the molten pool’s stoichiometry. Traditionally, regulating alloy components has relied on time-consuming phase diagram calculations. However, the synergistic effects of various factors, combined with the accumulation of material databases and the integration of ML in component design, have significantly improved the efficiency of materials development. ML has been particularly instrumental in customizing multicomponent alloys for AM applications. Two primary scenarios have emerged for leveraging ML to optimize AM properties. 

**Figure 4:**
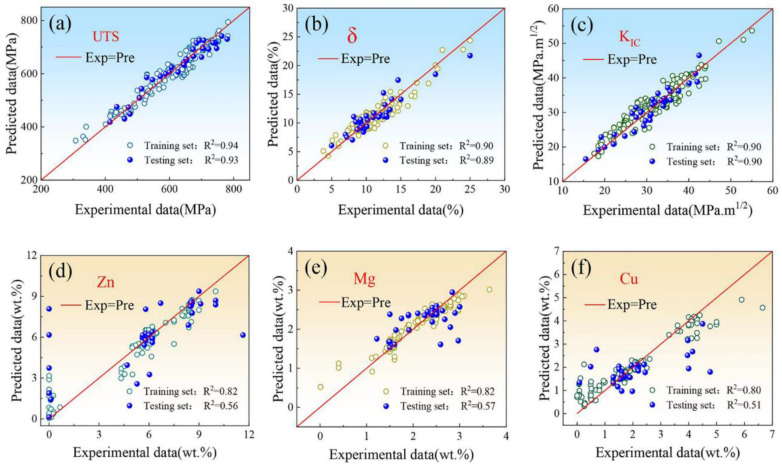
Training results of two neural network models’ composition to property model (C2P): (**a**) ultimate tensile strength (UTS); (**b**) elongation (δ); (**c**) fracture toughness (K_IC_); property to composition model (P2C); (**d**) Zn; (**e**) Mg; (**f**) Cu. (Adapted with permission from Ref. [87]. Copyright 2022 Elsevier).

The first one is to achieve grain refinement and inhibit the formation of brittle phases, thereby improving mechanical properties. For example, a machine learning design system (MLDS) [87] was established to rapidly design multiple alloy compositions meeting specific property requirements for aluminum alloys. The MLDS was established by two artificial neural network modules: the composition design (P2C) module, which takes attributes as input to predict alloy composition, and the performance prediction (C2P) module, which uses composition as input to predict the properties of the alloy. When the P2C module was utilized independently, three mechanical property indicators (ultra tensile strength, elongation, and fracture toughness) were employed to predict 11 alloy compositions, constituting a ‘dimension increasing fitting modeling’. This approach led to poor model convergence or a tendency to overfit, thereby compromising the reliability of composition design. In contrast, the C2P module is more suitable for the design of complex compositions. However, dependence on large-scale exhaustive exploration within the compositional space significantly diminishes the efficiency of the composition design. Recognizing the limitations of P2C and C2P modules, the MLDS combines these two models to expedite the selection of optimal compositions (Figure 4). When the desired target properties were specified, the P2C module was first utilized to generate candidate alloy compositions. These candidate compositions were then input into the C2P module, to predict the corresponding alloy properties. Subsequently, the predicted properties were compared with the target properties to assess their compliance with the predefined error tolerance thresholds. The high mass fraction of Mg and Zn elements promoted the formation of a high density of η’ phase, while the appropriate combination of microalloying elements such as Cr, Mn, Zr, and Ti refined the grain structure, thereby enhancing the mechanical properties of the alloy. 

The second application is to reduce cracks and defects in the AM process, thereby improving the overall printing quality. A notable design combines Scheil-Gulliver solidification simulations with ML analysis to design Fe-based alloys resistant to liquid-state cracking [88]. During the rapid cooling process inherent to PBF, the solubility of alloying elements decreases as the temperature declines, resulting in chemical segregation during the final stages of solidification. This segregation elevates the solidus temperature of the material, leading to the formation of solute-enriched regions with reduced melting temperatures, which may serve as potential crack initiation sites. Consequently, through strategic compositional design, the development of an alloy characterized by a stable single-phase microstructure and minimal chemical segregation will markedly diminish the propensity for solidification cracking. 50 Fe-Al-Cr alloys with varying Cr and Al compositions were selected as the target systems for compositional screening. A developed Matlab-ThermoCalc integration script was utilized to automate the compositional screening process. Upon completion of the analysis for all alloys, the solidification gradient (SG) and solidification range (SR) were derived from the solidification curves and utilized as training dataset for Gaussian process regression (GPR) model. The model analyzed the effect of alloying elements on solidification gradient and range, therefore provided the optimal composition (Fe-20Cr-7Al-4Mo-3Ni). The alloy was confirmed to be a single-phase material with low texture and negligible chemical segregation. This approach successfully prevented both solidification and liquefaction cracks during the printing process, demonstrating the effectiveness of ML-driven composition design.

### 4.4. ML in Geometry Design

AM enables the high-precision digital control necessary to create complex structures, making geometry design a focal point in recent AM research. ML-guided design of porous structural materials has shown tremendous potential in this regard. Typically, AM porous structures are designed by repeating a representative volume element (RVE) in a lattice configuration. ML-guided geometry design, however, reverses this process by deducing the optimal structure from desired properties. This approach overcomes the limitations of manual topological design by allowing the RVEs to be randomly arranged within a wide design space.

**Figure 5:**
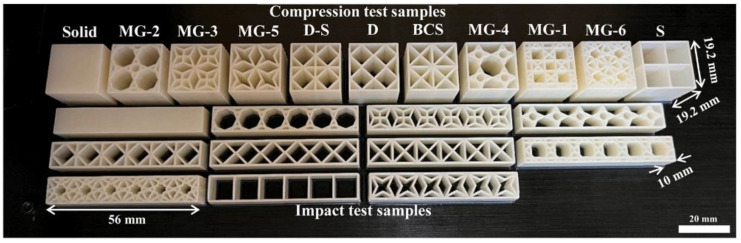
Lattice structure samples for compression tests oriented on the build plate: MG(1–6) are generated by the GAN model. The width of the impact test samples are all 10 mm. (Reprinted from Ref. [89]. Copyright 2024 Elsevier).

Yüksel et al. [89] proposed a deep learning-based GAN model to design lattice structures with enhanced mechanical properties. They initially utilized a parametric design approach to establish the training dataset for the GAN model. This parametric methodology ensures the continuity of lattice design and enhances the diversity of the dataset. After preliminary evaluation using a simulated annealing algorithm plugin, the training dataset was refined to retain only high-performance lattice structures, which were subsequently used for GAN model training to ensure that the generated structures also exhibit high performance. Six unit cell types generated by the GAN model were compared with four standard unit cells in terms of mechanical properties (Figure 5). The results demonstrated that the GAN-generated unit cells exhibited superior compressive properties, elongation rates, and enhanced energy absorption capabilities compared to the standard unit cells. This study provides a foundational framework for data-driven design methodologies in the development of porous structures.

Additionally, Iyer et al. [90] developed a producibility-aware topology optimization (PATO) framework to explore the design space of components fabricated by AM. By utilizing an attention-based U-Net architecture for surrogate modeling, the framework predicted shear strain index values, helping to fabricate defect-free specimens. The designs produced by PATO significantly reduced the maximum shear strain index (MSSI), resulting in crack-free parts.

### 4.5. ML in Processing Monitoring

**Figure 6:**
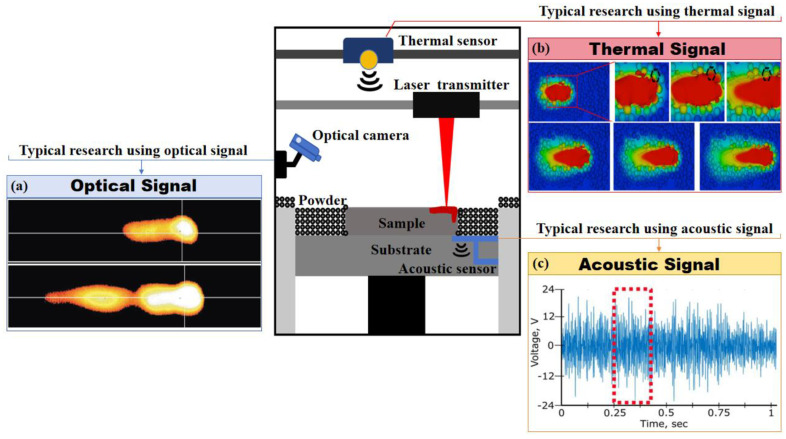
Typical signals for process monitoring in AM: (**a**) molten pool images of Fe-based alloy under different atmospheres. (Adapted with permission from Ref. [93]. Copyright 2006 Elsevier); (**b**) thermal distribution of single tracks. (Adapted with permission from Ref. [94]. Copyright 2017 Elsevier); (**c**) an example of a fragment of an acoustic emission signal that corresponds to a high quality layer produced with optimal process condition at medium energy density in AM stainless steel (79 J/mm^3^, 500 mm/s), the further analysis of acquired acoustic signals was performed using a running window (RW) which is schematically marked in red. (Adapted with permission from Ref. [75]. Copyright 2018 Elsevier).

AM is a complex process involving multiple interrelated physical phenomena. To enhance *in situ* control during processing, ML has been introduced for real-time monitoring and analysis. The primary goal of integrating ML into processing monitoring is to develop algorithmic models capable of providing real-time feedback and adjustments, ensuring high-quality prints. Various types of *in situ* signals are utilized for process monitoring in AM, and optical, thermal, and acoustic signals are the most extensively utilized (Figure 6a–c).

ML models have been developed to predict the likelihood of pore formation during AM by analyzing surface temperature data, using black body radiation and temporal characteristics as input variables [91]. The authors proposed that thermal history characteristics, including peak radiation and time-dependent cooling rates, are intrinsically linked to molten pool dynamics. They employed a customized infrared camera coupled with high-speed X-ray imaging to simultaneously monitor blackbody radiation from the sample surface, molten pool behavior, and pore formation processes. A predictive model based on a genetic algorithm was trained and developed by the thermal datasets. This model predicted the likelihood of pore formation under varying thermal history conditions, offering a tool for real-time control of PBF aimed at minimizing defect levels.

Furthermore, Zhu et al. [92] developed a defect prediction model by simulating fluid dynamics and interlayer fluid changes in the molten pool. The authors explored the use of Physics-Informed Neural Networks (PINNs) to predict temperature and molten pool fluid dynamics in metal AM processes. The input datasets for model training, including alloy properties, laser power, and scanning speed, were derived from a high-fidelity thermal-fluid finite element model. The prediction objectives encompassed the temperature and molten pool dynamics—specifically, melt flow velocity, pressure distribution, and temperature fields—during metal AM processes. Evaluation outcomes indicated that the PINN is capable of accurately predicting temperature and molten pool dynamics in metal AM processes, even with a relatively small volume of labeled data. 

Recent studies have also extensively explored several types of signals in AM processing monitoring, including optical signals from molten pool [93], thermal distribution of single printing track [92], and acoustic signals generated in the AM process [75]. The feasibility of printing quality prediction is demonstrated by establishing the ML model, which presented the influence of physical effects on the AM process.

### 4.6. ML in Parameter Optimization

AM parameter optimization often requires considerable manual intervention, particularly when introducing new compositions into an existing process scheme, leading to high costs. Recently, ML has been successfully applied to optimize AM parameters by defining parameter windows that yield high-density parts with minimal defects. ML input datasets, derived from small-scale experiments, have been used to model properties such as density [95], single-track printability [96], and surface morphology [97]. Several ML models have been developed to predict and optimize AM process parameters.

For example, an ML model based on small ensembles of decision trees (DTs) was built to predict ink-jetting behavior during the printing process [98]. A comprehensive dataset was assembled, comprising both laboratory and literature-derived data, to establish correlations between critical machine parameters (e.g., frequency, dwell voltage, and echo voltage, etc.) and droplet pattern formation on the substrate, across various materials. This dataset utilized a multitude of material and machine parameters as input variables to predict two continuous outputs—drop velocity and drop radius—as well as a discrete output, which is the jetting quality categorized into three levels: ’single drop’, ’no ejection’, or ’multiple drops’. These categories are collectively termed as ’drop behavior’. A composite regression model based on DTs was established and trained. This model demonstrated high predictive accuracy, achieving root mean square errors (RMSE) of 0.39 m/s for drop velocity and 2.12 μm for radius. Additionally, a three-layer deep neural network (DNN) model was established and classified drop behavior with 91.94% accuracy, offering an efficient alternative to traditional experimental methods.

**Figure 7:**
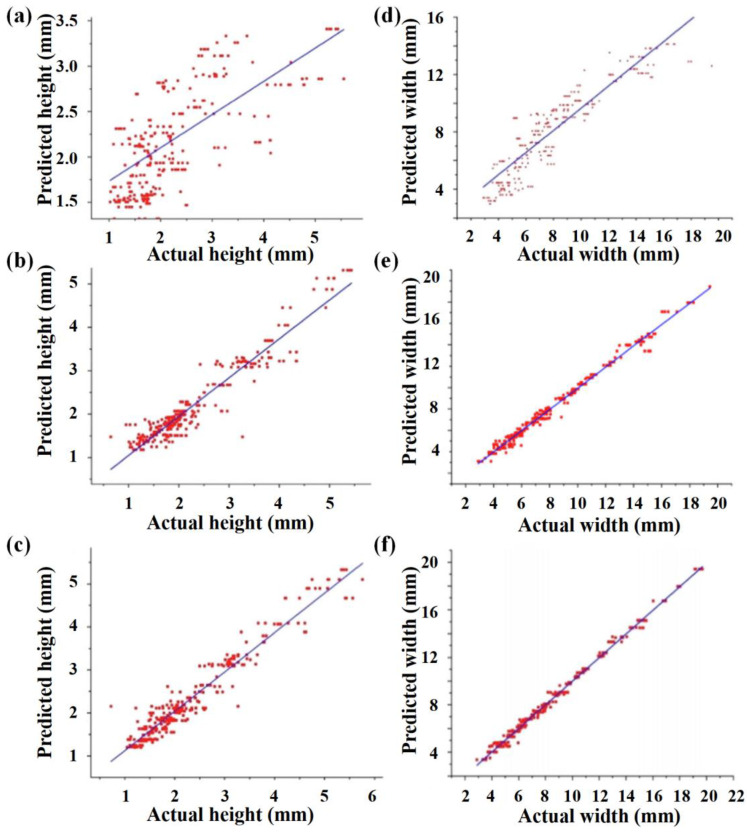
Actual vs. predicted bead height and width values graphs of different algorithms for WAAM 316L stainless steel: (**a**,**b**) support vector regression; (**c**,**d**) k-nearest neighborhood; (**e**,**f**) random forest. (Adapted with permission from Ref. [99]. Copyright 2023 Elsevier).

Another study developed the ML model with different algorithms to independently predict the bead height and width using the input variables in wire arc AM (WAAM) [99]. The random forest algorithm demonstrated the best fitness of data points (Figure 7). Performance analysis indicated that the random forest reached the highest predicting accuracy (96% accuracy for height and 99% accuracy for width) compared to k-nearest neighborhood and support vector regression. 

## 5. Machine Learning in AM Metallic Biomaterials

The AM process generally involves complex high-throughput calculations, including physical and thermal field changes. ML-guided component design, geometry design, process control, and parameter optimization have thus emerged as promising studies. 

The intricate requirements of metallic biomaterials—ranging from mechanical strength to biocompatibility—demand precise control over material composition, microstructure, and manufacturing processes. Traditional trial-and-error approaches in AM often struggle to meet these demands due to the complexity of multicomponent alloys, intricate geometries, and the dynamic nature of the AM process. By leveraging ML algorithms, researchers can optimize alloy compositions, predict process outcomes, and design complex geometries with unprecedented efficiency and accuracy. This integration of ML into AM workflows is reshaping the development of next-generation metallic biomaterials, enabling innovative solutions that were previously unattainable.

### 5.1. ML-Guided Component Design in AM Biomaterials

The composition of a material governs its microstructure and, consequently, its performance in AM. Current research on AM biomaterials has shifted from using fixed-grade alloys to focusing on optimizing multicomponent alloys, particularly for inert biomaterials. With significant progress already made in the composition design of Ti-based alloys and stainless steels, ML-guided approaches are now at the forefront of component optimization.

For example, a computational approach combining ML, phase diagrams calculations, and physical models has been proposed to design austenitic alloys [100]. These alloys primarily rely on Ni, Mn, and Co as key alloying elements to stabilize the face centered cubic (FCC) solid solution, making component design crucial. A multi-objective genetic algorithm (GA) model was developed to automate the component optimization process, efficiently identifying promising alloy compositions. Subsequent validation demonstrated the model’s effectiveness in AM applications. 

**Figure 8:**
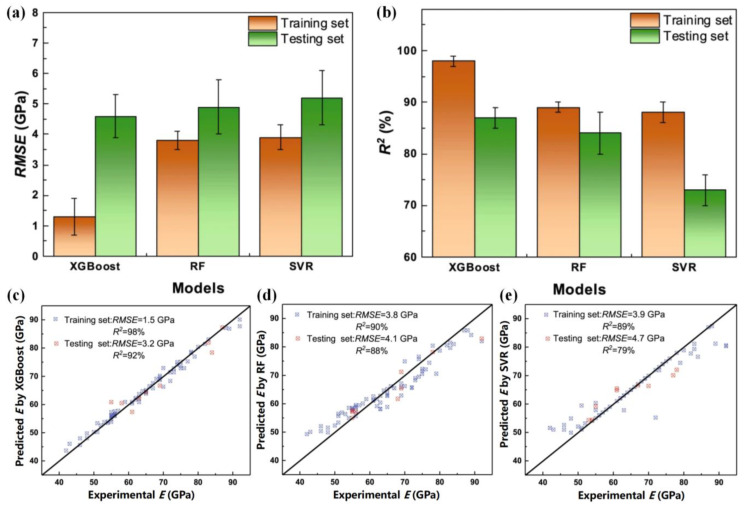
Statistical results on training set and testing set of ML models for *E* prediction of β-Ti alloys: (**a**) *RMSE*; (**b**) *R*^2^, and the experimental *E* values vs. the predicted values by the optimal model: (**c**) extreme gradient boosting; (**d**) random forest; (**e**) support vector regression. The blue points and red points represent the training set and the testing set, respectively. (Adapted with permission from Ref. [101]. Copyright 2020 Springer Nature).

Similarly, a GA model embedding the molybdenum equivalence and the cluster formula was employed to design low Young’s modulus (E) multicomponent β-Ti alloys [101]. The model predicted several new β-Ti alloys with specific E values by setting different objective functions. Experimental validation confirmed that the alloys achieved the desired properties, demonstrating the high prediction accuracy of the cluster-formula-embedded ML model (Figure 8c–e).

### 5.2. ML-Guided Geometry Design in AM Biomaterials

The design of biomaterials focuses not solely on maximizing load-bearing capacity, stiffness, or lifetime but on tailoring mechanical and degradation properties to meet specific biomedical requirements. This can be achieved by controlling the geometry of specimens, allowing for optimization based on the intended application.

An alternative approach using an unsupervised generative adversarial network (GAN) model has been reported for the development of novel, nature-inspired architected materials [102]. By using a dataset of leaf structures, a library of architected unit cells was generated to establish procedures for using the latent space for the transfer of information across manifestations in the directed design of 2D and 3D materials. To explore the structural interactions within the latent space, the model was retrained with a combined dataset of leaf images and trabecular bone images. This retraining resulted in unique microstructures with varying degrees of hierarchy, making them suitable for biomaterial applications.

**Figure 9:**
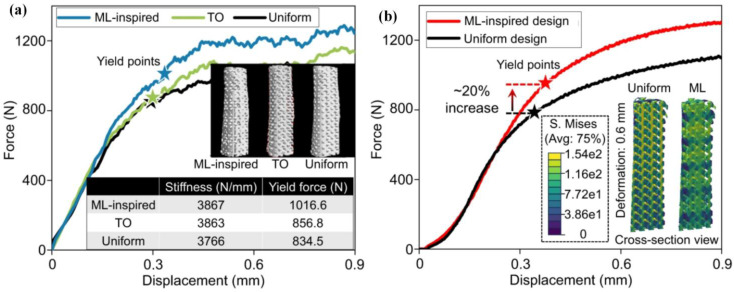
Anatomic bone fixation with ML-inspired design: (**a**) finite element method simulated displacement-force curves of ML-inspired, topology optimization (TO), and uniform designs; (**b**) experimental displacement-force curves of the ML-inspired design vs. uniform design. (Adapted with permission from Ref. [103]. Copyright 2023 Springer Nature).

Additionally, a data-efficient method employing generative architecture design was proposed for a high-dimensional, multi-property optimization of AM materials [103]. An overall method ‘GAD-MALL’ integrating generative architecture design (GAD) and Multi-objective active learning loop (MALL) was applied to a design porous structure to optimize the mechanical properties of bone grafting implants. GAD was employed to generate a set of architectures with unknown properties, utilizing gyroid units as model inputs to minimize complexity and optimize computational efficiency. Concurrently, the MALL system was implemented to evaluate the dataset generated by GAD. Through a recursive query finite element approach, MALL systematically identified architectures demonstrating enhanced performance. This ML-guided approach enabled the design of microscale heterogeneous architectures with improved strength and a biocompatible elastic modulus. The load-bearing capacity of implants inspired by ML-designed structures was significantly higher than those with uniform designs (Figure 9b).

### 5.3. ML-Guided Processing Control in AM Biomaterials

AM processes are inherently coupled with multiple complex physical phenomena, generating a variety of signals. ML models have been increasingly utilized to optimize the processing of biomaterials by collecting and analyzing these signals in real-time.

Zhang et al. [104] developed a computational framework based on the discrete element method (DEM) for preparing AM Ti-6Al-4V specimens. Using a back-propagation neural network (BP-NN) model, the relationship between powder layer parameters and spreading speed was established. Simulations of the Ti-6Al-4V powder diffusion process on a smooth lining revealed the impact of powder spreading on the surface roughness of specimens. The model demonstrated near-perfect regression, with low error rates and high R-values (R ≥ 0.98175).

In another study, a posture monitoring method was proposed for fault diagnosis in AM [105]. By installing several posture sensors in the printer, the attitude data under different conditions were collected. Fault diagnosis was performed using support vector machine (SVM) and binary classification models, with the SVM model achieving the highest accuracy (94.44%) when utilizing all channels of attitude monitoring data. 

**Figure 10:**
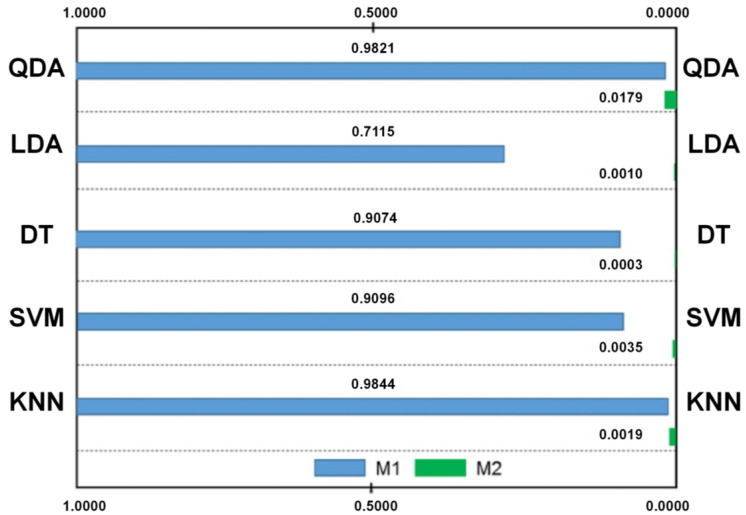
Comparing the accuracy measures for the classification methods with simple metrics when identify printing defects in AM Ti-6Al-4V specimens (QDA: quadratic discriminant analysis; LDA: linear discriminant analysis; DT: decision tree; SVM: support vector machine; KNN: K-Nearest Neighbor). (Adapted with permission from Ref. [106]. Copyright 2018 Elsevier).

For real-time porosity prediction, an ML-based morphological model was developed to analyze molten pool images and identify printing defects in AM Ti-6Al-4V specimens [106]. Two evaluation metrics were defined: M1, representing the probability of correctly predicting abnormal molten pools, and M2, representing the probability of incorrectly predicting porosity. Numerical experiments demonstrated that the morphological model, combined with supervised learning techniques, exhibited significant potential for real-time detection of microstructural anomalies during AM processes (Figure 10).

### 5.4. ML-Guided Parameter Optimization in AM Biomaterials

Traditional AM parameter optimization often relies on prior knowledge and subjective judgement, with optimal parameter windows typically determined through trial-and-error methods. This approach is both time-consuming and resource-intensive, leading to inevitable inefficiencies. However, the development of surrogate models linking parameters to material density has enabled ML-guided optimization, significantly reducing costs and improving efficiency. 

**Figure 11:**
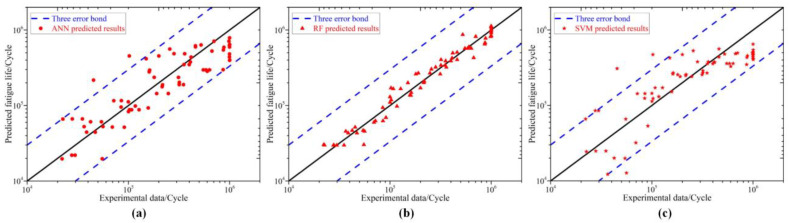
Variations in the predicted results by different ML models against the experimental data for AM SS316L under fatigue loads ranging from 145 to 660 MPa: (**a**) artificial neural network; (**b**) random forest; (**c**) support vector machine. (Adapted with permission from Ref. [107]. Copyright 2021 Elsevier).

Zhan et al. [107] developed a data-driven analysis platform based on continuum damage mechanics for AM SS316L. Using a comprehensive database, different ML models were trained and validated, with the random forest model achieving the highest accuracy in fatigue life prediction. The study revealed that the proportion of the training set significantly impacted prediction accuracy, with relative error decreasing rapidly as the training data increased (Figure 11).

Similarly, a numerical model based on an artificial neural network (ANN) was proposed to predict the tensile properties of SLM Ti-6Al-4V specimens and analyze the effects of process parameters [108]. The ANN model proved effective in handling nonlinear relationships, making it well-suited for parameter optimization. By optimizing process parameters, tensile strength was significantly improved (from 756.87 to 1537.67 MPa), demonstrating the substantial potential of ML-guided models in enhancing AM processes.

## 6. Other Properties

AM, when combined with advancements in ML algorithms, offers unprecedented potential to produce high-quality biomaterial components with customized properties. As the demand for tailored biomaterials continues to rise, ML-guided AM fabrication emerges as a promising solution to meet these evolving needs. Future research is expected to explore the application of more specialized ML algorithms in the AM of metallic biomaterials. To enhance the performance of ML models, hybrid approaches combining multiple algorithms can be utilized, customized to meet specific requirements, and incorporate diverse input and output data to evaluate comprehensive performance metrics. With the ongoing development and maturation of ML techniques, it is expected that the automated frameworks incorporating a variety of ML algorithms will be developed and extensively adopted for tasks including component design, structural optimization, and property prediction of AM metallic biomaterials.

While current research largely emphasizes process optimization, relatively few studies focus on applying ML to simulate the biological performance of biomaterials. For example, ion release experiments for biodegradable metals typically require up to 28 days of simulated immersion in body-fluid-like environments, with ion concentrations measured every two days. This process incurs significant time and economic costs. Introducing dynamic immersion conditions further complicates data collection and environmental control. Additionally, variables such as body fluid composition, flow, temperature, and pH significantly influence degradation performance, often resulting in notable discrepancies between *in vitro* simulations and *in vivo* degradation. This gap necessitates extensive and costly clinical testing. Antoniac et al. [109] highlighted these challenges, demonstrating that local tissue metabolism impacts the corrosion process, with *in vivo* corrosion rates being significantly lower than their *in vitro* counterparts. This underscores the urgent need for more scientifically robust *in vitro* testing methods. ML has achieved considerable success in modeling large corrosion datasets, facilitating accurate predictions of corrosion rates and the service life of metallic materials under various environmental conditions [110,111,112]. By selecting and optimizing appropriate algorithms, ML-guided corrosion simulation models can be developed to better align with the physiological conditions of individual patients. These models offer significant potential for use in *in vitro* corrosion simulations of biodegradable metallic materials, providing a reliable approach to enhance the accuracy of predictions and reduce the gap before clinical trials.

Another notable application is the integration of ML with computational modeling to optimize the biological properties of AM metallic materials. ML has been implemented for optimizing cell growth on AM biomaterials with empirically validated reliability. Wang et al. [113] proposed a data-driven morphology learning framework for topology optimization, which was subsequently applied to bone tissue scaffold design. This approach identified latent structural configurations with enhanced biological performance, followed by finite element analysis (FEA)-based mechanical optimization within the identified design space. Structures with high mechanical stiffness can be generated by this approach, which did not exist in original dataset. The human adipose-derived stem cells (hADSCs) cultured on the designed structures exhibited good cell adhesion. Compared to traditional scaffolds, the cell proliferation significantly enhanced, underscoring the suitability of the framework for advanced porous implant design. In the foreseeable future, ML is poised to significantly advance the application of AM metallic materials by overcoming current limitations. Specifically, ML is expected to enable the inverse design of porous structures optimized for specific biological performance metrics. This capability will address a wide range of application-specific requirements, thereby driving the development and widespread adoption of AM-fabricated metallic implants.

The challenge of generating sufficiently large and diverse datasets for studying physical and chemical properties remains a significant obstacle in training ML models. While ML-assisted AM of biomaterials holds considerable promise, it is essential to simultaneously advance the optimization of ML methodologies through a multifaceted approach:

**Data Generation through Advanced Techniques**: A promising strategy to address data scarcity involves the application of generative models. GANs, for instance, a model frequently discussed in this review, leverage the adversarial interaction between generative and discriminative models to synthesize data that closely approximates the distribution of the training set. This approach enables the augmentation of existing datasets, enhancing their diversity and increasing the volume of available training samples. Such improvements are critical for boosting model performance and addressing the limitations posed by insufficient data.

**Integration of Machine Learning with Physical Models**: To overcome the constraints of experimental data, integrating established physical models—such as those derived from mechanics and thermodynamics—with machine learning frameworks offers a viable solution. By incorporating a limited set of experimental data, optimized simulation models can be developed to generate large, high-fidelity datasets for ML applications. This approach not only enriches datasets but also enhances the interpretability and robustness of the resulting ML models by embedding physical laws and principles into the learning process.

**Application of Ensemble Learning Methods**: Another effective strategy involves the use of ensemble learning techniques, which aggregate predictions from multiple ML models to mitigate the biases inherent in individual model architectures. This method is particularly advantageous in scenarios with small or imbalanced datasets, as it improves both the accuracy and generalizability of the model’s predictions. By leveraging ensemble learning, more reliable and stable outcomes can be achieved in material design and optimization. 

By adopting these strategies, the field of ML-assisted AM can overcome data-related challenges, paving the way for more accurate, interpretable, and robust models in biomaterial research and development.

## 7. Concluding Remarks

ML holds significant potential in optimizing AM biomaterials and has garnered widespread attention in recent studies. This paper provides an overview of various AM processes and discusses the application of ML in AM biomaterials, focusing on four key areas of ML-guided AM process optimization:

1. Component design: AM biomaterials are increasingly moving towards multi-component alloys. By incorporating specific elements and adjusting the content of alloying elements, highly customized implants can be fabricated. This process requires consideration of atomic properties, thermodynamics, and phase kinetics. ML-guided component design models have proven beneficial in this context. ML models can be employed to design multi-component alloys and optimize alloy compositions according to specific practical requirements, enabling precise regulation of individual component ratios. This approach facilitates the achievement of desired microstructures, ultimately leading to the realization of targeted material properties. The combination of ML algorithms with high-throughput databases will drive the development of AM component screening in future research.
2. Structural design: designing specific porous structures allows for the customization of mechanical and biological properties, maximizing material utilization efficiency and reducing waste. ML-guided structural design facilitates the creation of intricate, complex porous implants, overcoming the limitations imposed by prior experience and imagination. ML-assisted structural design enables the reverse engineering of porous scaffolds, which are customized to meet the specific requirements of individual patients. This methodology allows for the precise regulation of mechanical properties, degradation rates, and biological performance, thereby facilitating the development of highly optimized and patient-specific structures.
3. Processing control: the multiple physical fields involved in the AM process directly influence the quality of the fabricated specimens. ML-guided models aim to establish real-time, *in situ* monitoring systems based on AM signals. ML decomposes and reduces the dimensionality of real-time physical signals. By employing clustering and regression algorithms, ML models establish correlations between signal characteristics and printing quality, thereby enabling automated *in situ* quality assessment of AM. By analyzing real-time feedback through processing monitoring, ML can facilitate dynamic adjustments to processing parameters, correct printing errors, and reduce costs.
4. Parameter optimization: recent studies have elucidated the linkages between process parameters and material properties in AM. Various AM process parameters can significantly affect the quality of the final product. ML is dedicated to incorporating multiple process parameters into optimization models, improving the efficiency and precision of AM processes. By leveraging process parameters and their associated characteristics—such as microstructure, mechanical properties, and printing quality—as input variables, ML models can be trained to autonomously identify optimal process parameters. This approach significantly reduces the time and economic costs typically associated with trial-and-error methods in parameter optimization. Furthermore, integrating optimization models with large-sample datasets can improve prediction accuracy and reveal counterintuitive, high-quality regions within the parameter space.

As ML is integrated throughout the entire AM process, from pre-processing to component and structural design, it significantly enhances the performance optimization of biomaterials. ML models simplify complex phase calculations and eliminate the trial-and-error approach commonly used in traditional component design. By automating component optimization, ML enables a more efficient exploration of material compositions and structural configurations, which is particularly crucial for multi-component alloys and custom implants. In structural design, ML’s ability to guide the creation of complex, optimized porous structures provides a powerful tool for improving mechanical and biological properties while minimizing material waste. Furthermore, ML-driven advancements in processing control and parameter optimization allow for real-time monitoring and dynamic adjustments to AM processes, ensuring that defects are minimized and final product quality is maximized.

Looking ahead, the integration of ML in AM biomaterials research holds immense potential for bridging the gap between *in vitro* and *in vivo* performance predictions. While much of the current focus remains on process optimization, future research could explore ML’s application in simulating the degradation behavior and optimizing biological performance of AM biomaterials, offering more accurate and cost-effective methods for pre-clinical testing. ML-guided simulations, combined with physical models, could significantly reduce the reliance on traditional, time-consuming clinical trials. The continued evolution of ML in AM is expected to further enhance the capabilities of additive manufacturing, driving the development of high-performance, customized biomaterials that can be efficiently produced for a wide range of medical applications.

## Figures and Tables

**Table 1:** Introduction of several commonly used supervised learning methods.

Method	Principle	Advantages
Decision Tree	A tree-like model is progressively constructed by recursively partitioning the dataset into smaller subsets. Each internal node represents a test on a feature or attribute, each branch denotes the outcome of the test, and each leaf node signifies a class (classification) or a value (regression).	Clear structure with low computational complexity for discrete and non-discrete data simultaneously.
Support Vector Machine	A hyperplane is identified to separate data points of different classes in a multidimensional space, with the objective of maximizing the margin between the hyperplane and the nearest data points.	Suitable for small datasets and high dimensional problems, demonstrating strong generalization capabilities and the ability to handle nonlinearity.
Convolutional Neural Network	A network is built for features extraction from high-dimensional input data through local receptive fields and weight sharing, well-suited for image feature extraction.	High computational efficiency in image processing with low overfitting.

**Table 2:** Introduction of three commonly used unsupervised learning methods.

Method	Principle	Advantages
K-Means Clustering	The dataset is partitioned into K clusters through iterative optimization, ensuring that data points within each cluster are as similar as possible, while data points across different clusters are as distinct as possible, thereby achieving effective data segmentation.	Fast and efficient to large-scale data processing.
Gaussian Mixture Models	The data are assumed to be generated from a mixture of multiple Gaussian distributions, with each Gaussian component representing an individual cluster. The model characterizes the underlying data distribution by estimating the parameters of these Gaussian components.	Capable of fitting data distributions with arbitrary shapes and effectively capture complex structures.
Auto Encoder	The encoder compresses the input data into a low-dimensional representation, which is then reconstructed into the original data by the decoder, achieving dimensionality reduction, feature extraction, and noise reduction.	Enhance data quality and usability while retaining critical information.

**Table 3:** Introduction of three commonly used semi-supervised learning methods.

Method	Principle	Advantages
Semi-supervised G-model	The distribution of data is learned from the utilization of generative models, such as Generative Adversarial Networks (GANs) or Variational Autoencoders (VAEs), and unlabeled data are leveraged to assist in classification tasks.	The integration of diverse generative models and classifiers can be employed to adapt to varying task requirements, thereby enhancing the flexibility and efficacy.
Self-training	The model’s predictions on unlabeled data (pseudo-labels) are utilized to augment the training set through an iterative process, thereby enhancing the model’s performance.	Cost-effective in terms of labeling and requires only a base model and a pseudo-label generation mechanism, which is straightforward to implement.
Co-training	Multiple models are trained by utilizing multiple views or feature sets of data, providing complementary information from multiple perspectives, thereby enhancing each model’s performance.	Low annotation cost and suitable for multimodal data (e.g., image, audio, and text).

## Data Availability

No new data were created or analyzed in this study. Data sharing is not applicable to this article.

## References

[1] Vock S., Kloeden B., Kirchner A., Weissgaerber T., Kieback B. (2019). Powders for powder bed fusion: A review. Prog. Addit. Manuf..

[2] Bär F., Berger L., Jauer L., Kurtuldu G., Schäublin R., Schleifenbaum J.H., Löffler J.F. (2019). Laser additive manufacturing of biodegradable magnesium alloy WE43: A detailed microstructure analysis. Acta Biomater..

[3] Jahr H., Li Y., Zhou J., Zadpoor A.A., Schröder K.-U. (2021). Additively Manufactured Absorbable Porous Metal Implants—Processing, Alloying and Corrosion Behavior. Front. Mater..

[4] Li P., Dai J., Li Y., Alexander D., Čapek J., Geis-Gerstorfer J., Wan G., Han J., Yu Z., Li A. (2024). Zinc based biodegradable metals for bone repair and regeneration: Bioactivity and molecular mechanisms. Mater. Today Bio.

[5] Li Y., Shi Y., Lu Y., Li X., Zhou J., Zadpoor A.A., Wang L. (2023). Additive manufacturing of vascular stents. Acta Biomater..

[6] Geetha M., Singh A.K., Asokamani R., Gogia A.K. (2009). Ti based biomaterials, the ultimate choice for orthopaedic implants—A review. Prog. Mater. Sci..

[7] Alexandrino L., Antunes L., Munhoz A., Filho A.P., Silva W. (2022). Mechanical and surface properties of Co-Cr alloy produced by additive manufacturing for removable partial denture frameworks. J. Prosthet. Dent..

[8] Zhang Y., Lv Z., Luo X., Qiang H., He J., Hou C., Li Y., Liu F., Wang L. (2024). Synergistic release of copper and lithium components in biodegradable zinc alloy for osteoimmunomodulation. Rare Met..

[9] Frattolin J., Roy R., Rajagopalan S., Walsh M., Yue S., Bertrand O.F., Mongrain R. (2019). A manufacturing and annealing protocol to develop a cold-sprayed Fe-316L stainless steel biodegradable stenting material. Acta Biomater..

[10] Li G., Zhang L., Wang L., Yuan G., Dai K., Pei J., Hao Y. (2017). Dual modulation of bone formation and resorption with zoledronic acid-loaded biodegradable magnesium alloy implants improves osteoporotic fracture healing: An in vitro and in vivo study. Acta Biomater..

[11] Wang Z. (2018). Study on Corrosion Properties of Medical Mg-Zn Alloy. Key Eng. Mater..

[12] Li Y., Jahr H., Pavanram P., Bobbert F.S.L., Puggi U., Zhang X.Y., Pouran B., Leeflang M.A., Weinans H., Zhou J. (2019). Additively manufactured functionally graded biodegradable porous iron. Acta Biomater..

[13] Jin Z., Zhang Z., Demir K., Gu G.X. (2020). Machine Learning for Advanced Additive Manufacturing. Matter.

[14] Meng L., McWilliams B., Jarosinski W., Park H.-Y., Jung Y.-G., Lee J., Zhang J. (2020). Machine Learning in Additive Manufacturing: A Review. J. Miner..

[15] LeCun Y., Bengio Y., Hinton G. (2015). Deep learning. Nature.

[16] Qu X., Yang H., Jia B., Yu Z., Zheng Y., Dai K. (2020). Biodegradable Zn-Cu alloys show antibacterial activity against MRSA bone infection by inhibiting pathogen adhesion and biofilm formation. Acta Biomater..

[17] Shi Y., Xu W., Che H., Zhao S., Chang W., Li X., Lu Y., Xue C., Zhang D., Wang L. (2024). The effect of topological design on the degradation behavior of additively manufactured porous zinc alloy. NPJ Mater. Degrad..

[18] Shi Y., Gao J., Li X., Tao Z., Huang C., Zhao S., Wu Y., Yang Y., Yang Y., Li Y. (2025). Additively manufactured biodegradable Zn metamaterials with tunable Poisson’s ratio and enhanced mechanical properties. Virtual Phys. Prototyp..

[19] Lin X., Huang W.D. (2015). Laser additive manufacturing of high-performance metal components. Sci. Sin..

[20] Plocher J., Panesar A. (2019). Review on design and structural optimisation in additive manufacturing: Towards next-generation lightweight structures. Mater. Des..

[21] Wang H., Zhang S., Wang X. (2009). Progress and Challenges of Laser Direct Manufacturing of Large Titanium Structural Components(Invited Paper). Chin. J. Lasers.

[22] Lorusso M., Trevisan F., Calignano F., Lombardi M., Manfredi D. (2020). A357 Alloy by LPBF for Industry Applications. Materials.

[23] Cortis D., Pilone D., Grazzi F., Broggiato G., Campana F., Orlandi D., Shinohara T., Planell O.S. (2024). Functionally graded material via L-PBF: Characterisation of multi-material junction between steels (AISI 316L/16MnCr5), copper (CuCrZr) and aluminium alloys (Al-Sc/AlSi10Mg). Prog. Addit. Manuf..

[24] Parvez M.M., Pan T., Chen Y., Karnati S., Newkirk J.W., Liou F. (2020). High Cycle Fatigue Performance of LPBF 304L Stainless Steel at Nominal and Optimized Parameters. Materials.

[25] Zhao R., Shmatok A., Fischer R.D., Prorok B.C. (2023). Linking alloy thermo-physical behavior to laser process parameters for density optimization in LPBF. Int. J. Adv. Manuf. Technol..

[26] Gu H., Wei C., Li L., Ryan M., Setchi R., Han Q., Qian L. (2021). Numerical and experimental study of molten pool behaviour and defect formation in multi-material and functionally graded materials laser powder bed fusion. Adv. Powder Technol..

[27] Sanchez S., Gaspard G., Hyde C.J., Ashcroft I.A., Ravi G.A., Clare A.T. (2022). On the thermomechanical aging of LPBF alloy 718. Mater. Sci. Eng. A.

[28] Ziaee M., Crane N.B. (2019). Binder jetting: A review of process, materials, and methods. Addit. Manuf..

[29] Jiang R., Monteil L., Kimes K., Mostafaei A., Chmielus M. (2021). Influence of powder type and binder saturation on binder jet 3D–printed and sintered Inconel 625 samples. Int. J. Adv. Manuf. Technol..

[30] Yanez-Sanchez S.I., Lennox M.D., Therriault D., Favis B.D., Tavares J.R. (2021). Model Approach for Binder Selection in Binder Jetting. Ind. Eng. Chem. Res..

[31] Butscher A., Bohner M., Roth C., Ernstberger A., Heuberger R., Doebelin N., von Rohr P.R., Müller R. (2012). Printability of calcium phosphate powders for three-dimensional printing of tissue engineering scaffolds. Acta Biomater..

[32] Mühler T., Gomes C., Ascheri M.E., Nicolaides D., Heinrich J.G., Günster J. (2015). Slurry-based powder beds for the selective laser sintering of silicate ceramics. J. Ceram. Sci. Technol..

[33] Mostafaei A., Elliott A.M., Barnes J.E., Li F., Tan W., Cramer C.L., Nandwana P., Chmielus M. (2021). Binder jet 3D printing—Process parameters, materials, properties, modeling, and challenges. Prog. Mater. Sci..

[34] Özel T., Shokri H., Loizeau R. (2023). A Review on Wire-Fed Directed Energy Deposition Based Metal Additive Manufacturing. J. Manuf. Mater. Process..

[35] Feenstra D.R., Banerjee R., Fraser H.L., Huang A., Molotnikov A., Birbilis N. (2021). Critical review of the state of the art in multi-material fabrication via directed energy deposition. Curr. Opin. Solid State Mater. Sci..

[36] Thompson S.M., Bian L., Shamsaei N., Yadollahi A. (2015). An overview of Direct Laser Deposition for additive manufacturing; Part I: Transport phenomena, modeling and diagnostics. Addit. Manuf..

[37] Chen J., Xie S., Huang H. (2024). In-situ powder mixing for laser-based directed energy deposition of functionally graded materials. Adv. Manuf..

[38] Woo W., Kim D., Kingston E.J., Luzin V., Salvemini F., Hill M.R. (2019). Effect of interlayers and scanning strategies on through-thickness residual stress distributions in additive manufactured ferritic-austenitic steel structure. Mater. Sci. Eng. A.

[39] Shin H., Lee J., Choi S., Lee S.W. (2023). Development of multi-defect diagnosis algorithm for the directed energy deposition (DED) process with in situ melt-pool monitoring. Int. J. Adv. Manuf. Technol..

[40] Jiang S., Zheng B., Svetlizky D., Valdevit L., Eliaz N., Lavernia E.J., Schoenung J.M. (2024). Thermal behavior of coated powder during directed energy deposition (DED). Materialia.

[41] Perumal V., Abueidda D., Koric S., Kontsos A. (2023). Temporal convolutional networks for data-driven thermal modeling of directed energy deposition. J. Manuf. Process..

[42] Yan L., Chen Y., Liou F. (2020). Additive manufacturing of functionally graded metallic materials using laser metal deposition. Addit. Manuf..

[43] Milošev I. (2017). From In Vitro to Retrieval Studies of Orthopedic Implants. Corrosion.

[44] Wong K., Scheinemann P. (2018). Additive manufactured metallic implants for orthopaedic applications. Sci. China Mater..

[45] Yan R., Luo D., Huang H., Li R., Yu N., Liu C., Hu M., Rong Q. (2018). Electron beam melting in the fabrication of three-dimensional mesh titanium mandibular prosthesis scaffold. Sci. Rep..

[46] Geng X., Li Y., Li F., Wang X., Zhang K., Liu Z., Tian H. (2020). A new 3D printing porous trabecular titanium metal acetabular cup for primary total hip arthroplasty: A minimum 2-year follow-up of 92 consecutive patients. J. Orthop. Surg. Res..

[47] Al J.Y.S. (2014). Physico-mechanical properties and prosthodontic applications of Co-Cr dental alloys: A review of the literature. J. Adv. Prosthodont..

[48] Chen Z., Phan M.A.L., Darvish K. (2017). Grain growth during selective laser melting of a Co-Cr-Mo Alloy. J. Mater. Sci..

[49] Barucca G., Santecchia E., Majni G., Girardin E., Mengucci P. (2015). Structural characterization of biomedical Co-Cr-Mo components produced by direct metal laser sintering. Mater. Sci. Eng. C.

[50] Kim K., Hwang J., Lee K. (2020). Effect of building direction on the mechanical anisotropy of biocompatible Co-Cr-Mo alloy manufactured by selective laser melting process. J. Alloys Compd..

[51] Xiang D., Wang P., Tan X., Chandra S., Wang C., Nai M.L.S., Tor S.B., Liu W., Liu E. (2019). Anisotropic microstructure and mechanical properties of additively manufactured Co-Cr-Mo alloy using selective electron beam melting for orthopedic implants. Mater. Sci. Eng..

[52] Dong X., Sun Q., Zhou Y., Qu Y., Shi H., Zhang B., Xu S., Liu W., Li N., Yan J. (2020). Influence of microstructure on corrosion behavior of biomedical Co-Cr-Mo-W alloy fabricated by selective laser melting. Corros. Sci..

[53] Brogini S., Sartori M., Giavaresi G., Cremascoli P., Alemani F., Bellini D., Martini L., Maglio M., Pagani S., Fini M. (2021). Osseointegration of additive manufacturing Ti-6Al-4V and Co-Cr-Mo Alloys, with and without Surface Functionalization with Hydroxyapatite and Type I Collagen. J. Mech. Behav. Biomed. Mater..

[54] Toh W.Q., Tan X., Sun Z., Liu E., Tor S.B., Chua C.K. Comparative study on tribological behavior of Ti-6Al-4V and Co-Cr-Mo samples additively manufactured with electron beam melting. Proceedings of the 2nd International Conference on Progress in Additive Manufacturing (Pro-AM 2016).

[55] Wang W., Yung K., Choy H., Xiao T., Cai Z. (2018). Effects of laser polishing on surface microstructure and corrosion resistance of additive manufactured CoCr Alloys. Appl. Surf. Sci..

[56] Rushing G.D., Goretsky M.J., Gustin T., Morales M., Kelly R.E., Nuss D. (2007). When it is not an infection: Metal allergy after the nuss procedure for repair of pectus excavatum. J. Pediatr. Surg..

[57] Karthika P., Olha B., Ming C., Madison R., Liam F., Jordan S., Richard S., Marcus T., Cameron C., Alex C. (2017). Metallic biomaterials: Current challenges and opportunities. Materials.

[58] Kruth J., Broucke B.V.D., Vaerenbergh J., Naert I. Rapid manufacturing of dental prostheses by means of selective laser sintering/melting. Proceedings of the Les 11èmes Assises Europeennes du Prototypage Rapide (AFPR) 2005.

[59] Wei S., Ma J., Xu L., Gu X., Ma X. (2020). Biodegradable materials for bone defect repair. Mil. Med. Res..

[60] Witte F. (2010). The History of biodegradable magnesium implants: A review. Acta Biomater..

[61] Hofstetter J., Martinelli E., Pogatscher S., Schmutz P., Povoden-Karadeniz E., Weinberg A.M., Uggowitzer P.J., Lffler J.F. (2015). Influence of Trace impurities on the in vitro and in vivo degradation of biodegradable Mg-5Zn-0.3Ca alloys. Acta Biomater..

[62] Liu J., Yin B., Sun Z., Wen P., Tian Y. (2021). Hot cracking in ZK60 magnesium alloy produced by laser powder bed fusion process. Mater. Lett..

[63] Karunakaran R., Ortgies S., Tamayol A., Bobaru F., Sealy M.P. (2020). Additive manufacturing of magnesium alloys. Bioact. Mater..

[64] Zeng Z., Salehi M., Kopp A., Xu S., Esmaily M., Birbilis N. (2022). Recent progress and perspectives in additive manufacturing of magnesium alloys. J. Magnes. Alloys.

[65] Carassus H., Guérin J.D., Morvan H., Haugou G., Sadat T., Guérard S., Markiewicz E. (2022). An Experimental investigation into influences of build orientation and specimen thickness on quasi-static and dynamic mechanical responses of selective laser melting 316L stainless steel. Mater. Sci. Eng. A.

[66] Muley S.V., Vidvans A.N., Chaudhari G.P., Udainiya S. (2016). An assessment of ultra fine grained 316L stainless steel for implant applications. Acta Biomater..

[67] Zhang S., Zhang X., Zhao C., Li J., Song Y., Xie C., Tao H., Zhang Y., He Y., Jiang Y. (2010). Research on an Mg-Zn Alloy as a degradable biomaterial. Acta Biomater..

[68] Zhu D., Su Y., Young M.L., Ma J., Zheng Y., Tang L. (2017). Biological responses and mechanisms of human bone marrow mesenchymal stem cells to Zn and Mg biomaterials. ACS Appl. Mater. Interfaces.

[69] Kannan M.B., Moore C., Saptarshi S., Somasundaram S., Rahuma M., Lopata A.L. (2017). Biocompatibility and biodegradation studies of a commercial zinc alloy for temporary mini-implant applications. Sci. Rep..

[70] Zhang D., Zhang L., Zhang X., Dai J., Zhao Y., Yang Q., Bai J., Xue F., Chu P.K., Chu C. (2024). Comparative investigation of the corrosion behavior of biomedical zinc alloys in oxygen-enriched simulated body fluid environments. Mater. Today Commun..

[71] Qin Y., Wen P., Voshage M., Chen Y., Schleifenbaum J.H. (2019). Additive manufacturing of biodegradable Zn-xWE43 porous scaffolds: Formation quality, microstructure and mechanical properties. Mater. Des..

[72] Zheng Y., Huang C., Li Y., Gao J., Yang Y., Zhao S., Che H., Yang Y., Yao S., Li W. (2024). Mimicking the mechanical properties of cortical bone with an additively manufactured biodegradable Zn-3Mg alloy. Acta Biomater..

[73] Foteinopoulos P., Papacharalampopoulos A., Angelopoulos K., Stavropoulos P. (2020). Development of a simulation approach for laser powder bed fusion based on scanning strategy selection. Int. J. Adv. Manuf. Technol..

[74] Matilainen V.-P., Piili H., Salminen A., Nyrhilä O. (2015). Preliminary investigation of keyhole phenomena during single layer fabrication in laser additive manufacturing of stainless steel. Phys. Procedia.

[75] Shevchik S.A., Kenel C., Leinenbach C., Wasmer K. (2018). Acoustic emission for in situ quality monitoring in additive manufacturing using spectral convolutional neural networks. Addit. Manuf..

[76] Moylan S., Whitenton E., Lane B., Slotwinski J. (2014). Infrared thermography for laser-based powder bed fusion additive manufacturing processes. AIP Conf. Proc..

[77] Chang C., Liao H., Yi L., Dai Y., Cox S.C., Yan M., Liu M., Yan X. (2023). Achieving ultra-high strength and ductility in Mg-9Al-1Zn-0.5Mn alloy via selective laser melting. Adv. Powder Mater..

[78] Chang C., Huang J., Yan X., Li Q., Liu M., Deng S., Gardan J., Bolot R., Chemkhi M., Liao H. (2020). Microstructure and mechanical deformation behavior of selective laser melted Ti6Al4V ELI alloy porous structures. Mater. Lett..

[79] Rahman M.S., Khomh F., Hamidi A., Cheng J., Antoniol G., Washizaki H. (2023). Machine learning application development: Practitioners’ insights. Softw. Qual. J..

[80] McCulloch W.S., Pitts W. (1943). A logical calculus of the ideas immanent in nervous activity. Bull. Math. Biophys..

[81] Minsky M., Papert S. (2017). Perceptrons, Reissue of the 1988 Expanded Edition with a New Foreword by Léon Bottou: An Introduction to Computational Geometry.

[82] Baumgartl H., Tomas J., Buettner R., Merkel M. (2020). A deep learning-based model for defect detection in laser-powder bed fusion using in-situ thermographic monitoring. Prog. Addit. Manuf..

[83] Zhang Y., Hong G.S., Ye D., Zhu K., Fuh J.Y.H. (2018). Extraction and evaluation of melt pool, plume, and spatter information for powder-bed fusion AM process monitoring. Mater. Des..

[84] Hertlein N., Deshpande S., Venugopal V., Kumar M., Anand S. (2020). Prediction of selective laser melting part quality using hybrid Bayesian network. Addit. Manuf..

[85] Snell R., Tammas-Williams S., Chechik L., Lyle A., Hernández-Nava E., Boig C., Panoutsos G., Todd I. (2020). Methods for rapid pore classification in metal additive manufacturing. J. Miner. Met. Mater. Soc..

[86] Okaro I.A., Jayasinghe S., Sutcliffe C., Black K., Paoletti P., Green P.L. (2019). Automatic fault detection for laser powder-bed fusion using semi-supervised machine learning. Addit. Manuf..

[87] Jiang L., Wang C., Fu H., Shen J., Zhang Z., Xie J. (2022). Discovery of aluminum alloys with ultra-strength and high-toughness via a property-oriented design strategy. J. Mater. Sci. Technol..

[88] Dovgyy B., Simonelli M., Pham M.-S. (2021). Alloy design against the solidification cracking in fusion additive manufacturing: An application to a FeCrAl alloy. Mater. Res. Lett..

[89] Yüksel N., Eren O., Börklü H.R., Sezer H.K. (2024). Mechanical properties of additively manufactured lattice structures designed by deep learning. Thin-Walled Struct..

[90] Iyer N., Mirzendehdel A.M., Raghavan S., Jiao Y., Ulu E., Behandish M., Nelaturi S., Robinson D. (2024). PATO: Producibility-aware topology optimization using deep learning for metal additive manufacturing. Int. J. Interact. Des. Manuf..

[91] Paulson N.H., Gould B., Wolff S.J., Stan M., Greco A.C. (2020). Correlations between thermal history and keyhole porosity in laser powder bed fusion. Addit. Manuf..

[92] Zhu Q., Liu Z., Yan J. (2021). Machine learning for metal additive manufacturing: Predicting temperature and melt pool fluid dynamics using physics-informed neural networks. Comput. Mech..

[93] Rombouts M., Kruth J.P., Froyen L., Mercelis P. (2006). Fundamentals of selective laser melting of alloyed steel powders. CIRP Ann..

[94] Yan W., Ge W., Qian Y., Lin S., Zhou B., Liu W.K., Lin F., Wagner G.J. (2017). Multi-physics modeling of single/multiple-track defect mechanisms in electron beam selective melting. Acta Mater..

[95] Silbernagel C., Aremu A., Ashcroft I. (2019). Using machine learning to aid in the parameter optimization process for metal-based additive manufacturing. Rapid Prototyp. J..

[96] Chen Y., Wang H., Wu Y., Wang H. (2020). Predicting the printability in selective laser melting with a supervised machine learning method. Materials.

[97] Özel T., Altay A., Kaftanoğlu B., Leach R., Senin N., Donmez A. (2019). Focus variation measurement and prediction of surface texture parameters using machine learning in laser powder bed fusion. J. Manuf. Sci. Eng..

[98] Brishty F.P., Urner R., Grau G. (2022). Machine learning based data-driven inkjet printed electronics: Jetting prediction for novel inks. Flex. Print. Electron..

[99] Sharma R., Raj Paul A., Mukherjee M., Vadali S.R.K., Kumar Singh R., Kumar Sharma A. (2023). Forecasting of process parameters using machine learning techniques for wire arc additive manufacturing process. Mater. Today Proc..

[100] Assi M., Favre J., Brykala M., Tancret F., Fraczkiewicz A. (2024). Design and assessment of an austenitic stainless alloy for laser powder bed additive manufacturing. Appl. Sci..

[101] Yang F., Li Z., Wang Q., Jiang B., Yan B., Zhang P., Xu W., Dong C., Liaw P.K. (2020). Cluster-formula-embedded machine learning for design of multicomponent β-Ti alloys with low Young’s modulus. NPJ Comput. Mater..

[102] Shen S.C.-Y., Buehler M.J. (2022). Nature-inspired architected materials using unsupervised deep learning. Commun. Eng..

[103] Peng B., Wei Y., Qin Y., Dai J., Li Y., Liu A., Tian Y., Han L., Zheng Y., Wen P. (2023). Machine learning-enabled constrained multi-objective design of architected materials. Nat. Commun..

[104] Zhang W., Desai P. (2017). Machine learning-enabled powder spreading process map for metal additive manufacturing. Solid Free. Fabr. Symp..

[105] He K., Yang Z., Bai Y., Long J., Li C. (2018). Intelligent fault diagnosis of Delta 3D printers using attitude sensors based on support vector machines. Sensors.

[106] Khanzadeh M., Chowdhury S., Marufuzzaman M., Tschopp M.A., Bian L. (2018). Porosity prediction: Supervised-learning of thermal history for direct laser deposition. J. Manuf. Syst..

[107] Zhan Z., Li H. (2021). Machine learning-based fatigue life prediction with effects of additive manufacturing process parameters for printed SS 316L. Int. J. Fatigue.

[108] Khorasani A.M., Gibson I., Ghaderi A., Mohammed M.I. (2019). Investigation on the effect of heat treatment and process parameters on the tensile behavior of SLM Ti-6Al-4V parts. Int. J. Adv. Manuf. Technol..

[109] Antoniac I., Adam R., Biță A., Miculescu M., Trante O., Petrescu I.M., Pogărășteanu M. (2021). Comparative assessment of in vitro and in vivo biodegradation of Mg-1Ca magnesium alloys for orthopedic applications. Materials.

[110] Wang H., Han E., Ke W. (2006). Predictive model for atmospheric corrosion of aluminium alloy by artificial neural network. J. Chin. Soc. Corros. Prot..

[111] Fu Z., Fu D., Li X. Atmospheric corrosion modelling with SVM based feature selection. Proceedings of the 2009 International Conference on Computational Intelligence and Software Engineering.

[112] Yan L., Diao Y., Lang Z., Gao K. (2020). Corrosion rate prediction and influencing factors evaluation of low-alloy steels in marine atmosphere using machine learning approach. Sci. Technol. Adv. Mater..

[113] Wang W., Hou Y., Su R., Wang W., Wang C.C.L. (2024). Simultaneous Topology Optimization of Differentiable and Non-Differentiable Objectives via Morphology Learning: Stiffness and Cell Growth on Scaffold. arXiv.

